# First-line single-agent panitumumab in frail elderly patients with wild-type RAS unresectable colorectal cancer: a phase II study protocol OGSG 1602

**DOI:** 10.1186/s12885-019-5821-z

**Published:** 2019-06-25

**Authors:** Tetsuji Terazawa, Takeshi Kato, Masahiro Goto, Daisuke Sakai, Yukinori Kurokawa, Toshio Shimokawa, Yoshihiro Matsubara, Taroh Satoh

**Affiliations:** 1 Cancer Chemotherapy Center, Osaka medical college, 2-7, Daigaku-machi, Takatsuki, Osaka, 569-8686 Japan; 2 Department of Gastroenterological Surgery, Osaka National Hospital, 2-1-14 Hoenzaka, Chuo-ku, Osaka, 540-0006 Japan; 3 Department of Frontier Science for Cancer and Chemotherapy, Osaka University Graduate School of Medicine, 2-15 Yamadaoka, Suita, Osaka, 565-0871 Japan; 4 Department of Gastroenterological Surgery, Osaka University Graduate School of Medicine, 2-15 Yamadaoka, Suita, Osaka, 565-0871 Japan; 5 Clinical Study Support Center, Wakayama Medical University, 811-1 Kimiidera, Wakayama, 641-8509 Japan; 6 Biostatistical Research Association, 2-22-10-A411 Kamishinden, Toyonaka, Osaka, 560-0085 Japan

**Keywords:** Colorectal cancer, Frail patient, Panitumumab, Clinical trial-trial design

## Abstract

**Background:**

A cytotoxic chemotherapeutic regimen is not routinely recommended for frail elderly patients with unresectable colorectal cancer (CRC) because of susceptibility to treatment. Panitumumab is a monoclonal antibody targeting the epidermal growth factor receptor (EGFR). Use of panitumumab as first-line therapy is expected to be well tolerated and to improve survival rates, even in patients who are not eligible for intensive chemotherapy. However, the efficacy and safety of panitumumab as the first-line therapy for the frail elderly patients with unresectable CRC have not been yet studied.

**Methods:**

We plan to conduct a prospective multi-center phase II study. Patients with wild-type RAS unresectable CRC aged ≥76 years or ≥ 65 who are not considered eligible for intensive chemotherapy will be included in the study. A total of 36 patients will be enrolled from Osaka Gastrointestinal Cancer Chemotherapy Study Group for over 2 years. Panitumumab 6 mg/kg IV infusion will be administered every 2 weeks. The purpose of this trial is to assess the efficacy of panitumumab as first-line therapy for patients with unresectable CRC. The primary endpoint is to determine the disease control rate. Secondary endpoints include progression-free survival, overall survival, response rate, time to treatment failure, and the incidence of grade 3/4 toxicities.

**Discussion:**

This is a prospective phase II trial assessing the efficacy of panitumumab monotherapy in the elderly patients with wild-type RAS unresectable CRC.

**Trial registration:**

The ethics committee of the Osaka Medical College approved this study on November 7, 2016. The trial registration number of the government was UMIN000024528 on December 1, 2016. It was registered prospectively (the day of enrollment of the first participant was February 9, 2017).

## Background

Advanced colorectal cancer (CRC) is the second most common cause of death from cancer in the world, after lung cancer [1, 2]. The elderly patients constitute more than half of the CRC cases and their prevalence increasing continuously [3]. A similar tend is observed in Japan; CRC is the second most common cause of death from cancer, with more than half of patients aged > 75 years [4, 5].

Treatment strategies for advanced CRC have been developed over the last decades; consequently, the median overall survival (OS) has now reached 30 months [6]. However, the frail elderly patients have never been the subjects of the clinical trials investigating these treatment strategies [7]. A pivotal trial in frail patients with CRC demonstrated that single-agent fluoropyrimidines were favorable to progression-free survival (PFS) and resulted in a better quality of life than intensive chemotherapy that included oxaliplatin [8]. Furthermore, the combination of irinotecan and fluoropyrimidines did not show a significant improvement in PFS compared with fluoropyrimidines alone [9]. Thus, intensive chemotherapy should not be routinely recommended for the frail elderly patients with unresectable CRC. Combination therapy with 5-fluorouracil and bevacizumab has been demonstrated to be well tolerated and effective. In a phase III AVEX trial in elderly patients, a combination of bevacizumab and capecitabine was shown to be superior to capecitabine alone in improving PFS, the primary endpoint of the study [10]. Based on the AVEX trial, the combination of bevacizumab and capecitabine is considered a standard therapy for elderly patients with unresectable CRC. However, even single-agent fluoropyrimidines often have undesirable effects, including fatigue, anorexia, gastrointestinal toxicities, or hematologic toxicity. As is often the case with frail elderly patients, a cytotoxic regimen is not routinely recommended because of their susceptibility to treatment.

Panitumumab is an epidermal growth factor receptor (EGFR)-inhibiting monoclonal antibody. Use of panitumumab as first-line therapy is expected to be well tolerated and to improve the survival rates even in patients with wild type (WT) RAS CRC who are not considered eligible for intensive chemotherapy [11, 12]. The presence of WT RAS, including KRAS and NRAS*,* is predictive of the effectiveness of anti-EGFR monoclonal antibodies [13, 14]. Panitumumab-related toxicities, including skin toxicity or hypomagnesemia, require management; however, panitumumab treatment was rarely found to cause cytotoxicity, including fatigue, appetite loss, or neutropenia. Thus, panitumumab may be suitable for the frail elderly patients [11, 12]. In addition, panitumumab is administered every 2 weeks in contrast to cetuximab, another EGFR-inhibiting monoclonal antibody, which is administered weekly. In a phase II trial in Spain, Sastre et al. investigated panitumumab as first-line therapy in frail elderly patients (≥70-year, PS 0–2) with WT KRAS unresectable CRC [11]. The median OS and progression free survival (PFS) were 7.1 months (95% CI, 5.0–12.3) and 4.3 months (95% CI, 2.8–6.4), respectively. Pietrantonia et al. also reported a retrospective study on the efficacy and the safety of panitumumab in the frail elderly patients with WT RAS and WT BRAF [12]. Although 75% of patients received panitumumab as second-line therapy, the median PFS and OS were 6.4 months (95% CI, 4.9–8) and 14.3 months (95% CI, 10.9–17.7), respectively [12].

The efficacy of panitumumab as first-line therapy has not been investigated in patients with WT RAS who are not eligible for intensive chemotherapy. There is no clear definition of frail elderly patients, however, the World Health Organization defined the elderly as individuals aged ≥65 years. Further, the Japanese government has redefined the term “elderly” as those individuals aged ≥75 years [15]. Thus, we will conduct a phase II trial on the efficacy of panitumumab in the frail elderly patients aged ≥76 years or ≥ 65 years who are not eligible for intensive chemotherapy. The results of this trial will affect the treatment strategy of CRC.

## Methods/design

The Osaka Gastrointestinal Cancer Chemotherapy Study Group (OGSG) Protocol Review Committee approved this study protocol on 18 October 2016. Patient enrollment began on February 9, 2017. Approval was obtained from the Institutional Review Board before starting patient accrual at each institution. This trial was registered at the UMIN Clinical Trials Registry as UMIN000024528 on December 1, 2016. The study is being conducted according to the guidelines of the Declaration of Helsinki and the International Conference on Harmonization E6 Good Clinical Practice. The ethical committee or institutional review committee at each site approved the protocol before the initiation of the study. All patients are required to sign a written informed consent.

### Endpoints

#### Primary endpoint

The primary endpoint is to determine the disease control rate (DCR), defined as a proportion of best overall response of complete response (CR), partial response (PR), or stable disease (SD). Response will be assessed based to the Response Evaluation Criteria in Solid Tumors (RECIST) version 1.1 [16].

#### Secondary endpoint

Secondary endpoints include determining OS (time from enrollment until death from any cause), PFS (time from enrollment until documented progressive disease or death from any cause), and response rate. Time to treatment failure and the incidence of grade 3/4 toxicities were also included as secondary endpoints. Adverse events are being graded according to the Common Terminology Criteria for Adverse Events (Japanese edition, JCOG version v4.03).

### Eligibility criteria

#### Inclusion criteria

1. Patients with histologically confirmed CRC who are not eligible for curative surgical resection
2. Patients with WT RAS
3. Patients with unresectable CRC who have received no previous systemic chemotherapy. The patients who relapsed in ≥6 months from the end of neoadjuvant or adjuvant chemotherapy are also included.
4. Patients aged ≥76 or ≥ 65 years who were not considered eligible for intensive chemotherapy by the treating physician.
5. Measurable disease according to the modified Response Evaluation Criteria In Solid Tumors (mRECIST) criteria (version 1.1).
6. Adequate organ function according to the following laboratory values obtained within 14 days before enrollment: neutrophil count, ≥1500/mm^3^; hemoglobin, ≥9 g/dL; platelet count, ≥10 × 10^4^/mm^3^; aspartate transaminase and alanine aminotransferase, ≤100 IU/L (in the presence of liver metastasis, ≤200 IU/L); total bilirubin, ≤2 mg/dL; creatinine clearance, ≤30 mL/min.
7. Life expectancy ≥90 days from enrollment
8. Written informed consent before study-specific screening procedure
9. Patients who did not previously receive treatment with anti-EGFR antibody

#### Exclusion criteria

1. Uncontrolled diarrhea
2. Symptomatic interstitial pneumonia or pulmonary fibrosis
3. Previous palliative radiation therapy for bone metastasis or brain metastasis within 2 weeks
4. History of other malignancy with a disease-free interval < 1 year (other than curatively treated cutaneous basal cell carcinoma, curatively treated carcinoma in situ of the cervix, and gastroenterological cancer confirmed to be cured by endoscopic mucosal resection)
5. Active infections
6. Serious complications: gastrointestinal bleeding, symptomatic heart disease (including unstable angina, myocardial infarction, and heart failure), and uncontrolled diabetes mellitus
7. History of serious anaphylaxis
8. Requirement of continuous treatment with systematic steroids
9. Psychiatric disability that would preclude study compliance
10. Positive for Hepatitis B surface antigen
11. Otherwise determined by the investigator to be unsuitable for participation in the study

### Treatment

Panitumumab 6 mg/kg IV infusion will be administered every 2 weeks. Patients will receive treatment until progressive disease, unacceptable toxicity, patient withdrawal/physician decision, or planned conversion surgery with the intention of curative resection.

After the second cycle, the protocol treatment will be started if skin toxicities (acne, dry skin, nail changes) are ≤ grade 2 and hypomagnesemia is ≤ grade 1 (Table 1) on day 1 of the cycle or the day before the scheduled date. If treatment cannot be started within 28 days, the patients will be withdrawn from the study. If there is grade 3 skin toxicities or hypomagnesemia (Table 2), the dose will be reduced (Table 3).

**Table 1:** Criteria for starting treatment after second cycle

	Criteria for starting treatment
Skin toxicities^a^	Grade ≦2
Infusion reaction	Grade≦1
Hypomagnesaemia	Grade ≦2
Nausea vomiting diarrhea	Grade ≦2
Anemia	Hb≧8.0 g/dL
Thrombocytopenia	≧50,000 /m^3^

^a^acne, dry skin, nail changes, etc.

**Table 2:** Adverse Events Requiring Dose Reduction

	Grade	The modification from next cycle
Skin toxicities^a^	≧3	Hold dose of treatment and One level dose down
Hypomagnesaemia	≧3	
Pulmonary fibrosis	≧2	Withdrawal from study
Infusion reaction	≧3	Hold dose of treatment

^a^acne, dry skin, nail changes, etc.

**Table 3:** Dose reduction levels

Level	Panitumumab
0	6 mg/Kg
-1	4.8 mg/Kg
-2	3.6 mg/Kg

### Study design and statistical considerations

The aim of the OGSG1602 phase II study is to assess the efficacy of panitumumab as first-line therapy for patients with WT RAS CRC who are not eligible for intensive chemotherapy. Therefore, we decided that the primary endpoint is the DCR and secondary endpoints are PFS, OS, response rate, time to treatment failure, and the incidence of grade 3/4 toxicities. The DCR is assessed by best response. An independent review committee assess efficacy. Based on the AVEX trial and the Spanish phase II study conducted by Sastre et al., the null hypothesis is “DCR is 45%,” and the alternative hypothesis is “DCR is >70%” [10, 11]; this will be assessed using an exact *p*-value of 0.05 and a power of 0.90 based on the Clopper-Pearson method. Thus, the sample size is 33. The total sample size is set to 36 to account for deviation. More than 22 events of DCR are needed for rejecting the null hypothesis. All statistical analyses will be conducted at the OGSG Data Center.

### Monitoring

The Data and Safety Monitoring Committee (DSMC) of the OGSG will independently review the efficacy and safety data obtained from the present study. On the basis of monitoring, the DSMC will consider the early termination of a treatment regimen during the study and a modification of the study protocol. Protocol compliance, safety, and on-schedule study progress will also be monitored by the DSMC. The monitoring will be performed annually.

## Discussion

The present study is the first prospective trial on the efficacy and safety of panitumumab as first-line therapy for patients with WT RAS aged ≥76 or ≥ 65 years who are not considered eligible for intensive chemotherapy. Although, for the frail elderly patients with unresectable CRC, combination therapy with 5-fluorouracil and bevacizumab is a standard care, a cytotoxic regimen is not routinely recommended because of the lack of tolerability. However, the use of panitumumab is expected to be well tolerated and to provide prolonged survival benefit. The present study has some limitations. First, the BRAF mutation is not excluded although the examination of BRAF status was approved in August 2018 in Japan. Second, there is no consideration of tumor location; this is because the efficacy of anti-EGFR monoclonal antibody in right-sided colon cancer was first reported in ASCO 2016 while the protocol of the present study was waiting approval from the ethics committee. Third, the primary endpoint of this study is DCR, although the primary endpoint of a phase II study is generally the response rate. Panitumumab rarely causes severe toxicities, including fatigue or anorexia, especially in frail elderly patients; therefore, panitumumab monotherapy is considered less toxic than capecitabine and bevacizumab combination therapy. Furthermore, the response rate was not high (19%) despite of 74% DCR and a favorable OS with capecitabine and bevacizumab therapy; however, Pietrantonio et al. reported that response rate of panitumumab as first-line therapy was 40% and DCR was 70% [10, 12]. Hence, we decide that the primary endpoint will be DCR. Additionally, in the present study, sarcopenia will be examined as a geriatric assessment, although translational analyses and examination of quality of life are not planned. The findings of the present study will help to establish anti-EGFR monoclonal antibody as the first line of treatment for frail elderly patients with unresectable CRC.

## Data Availability

The datasets used and/or analyzed during the current study are available from the corresponding author on reasonable request.
